# SoLAT (Sorafenib Lenvatinib alternating treatment): a new treatment protocol with alternating Sorafenib and Lenvatinib for refractory thyroid Cancer

**DOI:** 10.1186/s12885-018-4854-z

**Published:** 2018-10-04

**Authors:** Soo Young Kim, Seok-Mo Kim, Ho-Jin Chang, Bup-Woo Kim, Yong Sang Lee, Cheong Soo Park, Ki Cheong Park, Hang-Seok Chang

**Affiliations:** 1 Thyroid Cancer Center, Gangnam Severance Hospital, Department of Surgery, Yonsei University College of Medicine, Seoul, 120-720 South Korea; 2 Department of Surgery, Yonsei University College of Medicine, 50-1, Yonsei-ro, Seodaemun-gu, Seoul, 120-752 South Korea; 3 Yonsei Institute of Refractory Thyroid Endocrine Cancer, Yonsei University, Seoul, South Korea

**Keywords:** Papillary thyroid cancer, Lenvatinib, Sorafenib, EMT, FGF signaling, TKI

## Abstract

**Background:**

In the last decade, several tyrosine kinase inhibitors (TKIs), which disrupt pathways involved in the proliferation and tumorigenesis of thyroid cancer, have been extensively studied. Two different TKIs, lenvatinib and sorafenib, were recently approved by both the US FDA and European Medicine Agency. Until date, the duration of the TKI response is not sufficient and resistance eventually occurs. The goal of this study was to investigate a new treatment protocol, SoLAT, using sorafenib and lenvatinib alternatively on refractory thyroid cancer.

**Methods:**

Patient-derived aggressive papillary thyroid cancer (PTC) cell lines from patients with biochemical and histologically proven aggressive RAI-refractory papillary thyroid cancer were exposed to sorafenib and lenvatinib alternatively. Human thyroid cancer cell xenografts were obtained by injecting patient-derived aggressive PTC cell lines into the flank of female BALB/c nude mice. Tumor-bearing mice were treated with sorafenib and lenvatinib alternatively. Cell viability assay, immunofluorescence analysis, confocal imaging, immunoblot analysis, flow cytometry analysis of cell cycle and a tube formation assay were performed.

**Results:**

SoLAT was more effective for advanced PTC cell lines than individual treatment. Immunoblot analysis showed that SoLAT markedly increased levels of cell cycle inhibitors (p53 and p21), and pro-apoptotic factors (Apaf-1 and cleaved caspase 3) and decreased levels of positive cell cycle regulators (cyclin D1, CDK4, CDK6) and anti-apoptotic factors (p-NFκB, Bcl-2). Increased sub-G_0_/G_1_ population was observed in the SoLAT group, leading to apoptosis, cell cycle arrest, and strong inhibition of advanced PTC cell viability. SoLAT reduced the level of EMT markers such as vimentin, E-cadherin, Snail and Zeb1 by FGFR inhibition. In the xenograft model, individual treatment with sorafenib or lenvatinib did not markedly suppress patient-derived aggressive PTC cell xenograft tumors, whereas SoLAT significantly suppressed the proliferation of these tumors.

**Conclusions:**

SoLAT was more effective than individual treatment with sorafenib or lenvatinib in inhibiting PTC progression by inducing cell cycle arrest. Studies using both in vitro cell culture and an in vivo xenograft model provided evidence of tumor shrinkage with SoLAT. We suggest that these effects may be due to reduced EMT-mediated drug resistance in the aggressive PTC model.

## Background

Thyroid cancer accounts for more than 90% of all endocrine cancers and is the most common endocrine malignancy, as its incidence has increased over the last three decades [1]. Thyroid cancer is distinguished into well-differentiated, poorly differentiated, and anaplastic thyroid cancer based on cell differentiation characteristics and the ability to maintain follicular cell features. Differentiated thyroid cancer (DTC) is the most common thyroid cancer, representing more than 90% of all thyroid carcinomas. DTC is characterized by papillary and follicular histological subtypes [2, 3]. However, advanced cancer subtypes, including anaplastic thyroid cancer (ATC), have poor prognosis due to resistance to treatment and aggressive behavior [4], with total median survival of only few months [5]. Recently, novel targeted therapies have increased the lifespan of cancer patients. Kinase inhibitors are recommended for treating radioactive iodine (RAI)-refractory differentiated thyroid cancer (DTC) patients with metastatic, rapidly progressive, symptomatic, and/or imminently threatening disease that is not otherwise amenable to local control using alternative approaches [6]. Nevertheless, this has not been the case for patients with advanced cancer subtypes. Recent studies have revealed molecules and mechanisms that are closely connected to poor clinical outcomes in advanced thyroid cancer [7, 8]. Among these mechanisms, we concentrated on the epithelial-mesenchymal transition (EMT) and EMT-induced drug resistance of cancer stem cells (CSCs) as one of the probable reasons for the poor clinical results [9, 10]. EMT of cancer cells not only induces metastasis, but also contributes to drug resistance [10–12]. Therefore, it is necessary to determine the specific molecular changes or mechanisms of thyroid carcinogenesis to overcome the depressing outcome associated with advanced thyroid cancer.

Sorafenib was the first tyrosine kinase inhibitor tested in a phase III trial and was approved for the treatment of metastatic DTC in 2013. Patients with progressive RAI-refractory DTC treated with oral sorafenib showed improved progression-free survival compared to patients receiving placebo [13]. Lenvatinib was tested in RAI-refractory DTC patients in a phase III trial and was approved for use in RAI-resistant metastatic DTC in 2015 [14, 15]. The most important difference between lenvatinib and other drugs is its ability to inhibit fibroblast growth factor receptor 1 (FGFR1), making it an effective drug for cases with resistance to vascular endothelial growth factor/receptor (VEGF/R) inhibitors [16]. In reality, patients who were treated successively with sorafenib and lenvatinib or vice versa did not have any further treatment option. In metastatic renal cell cancer, the sequential use of two tyrosine kinase inhibitors was tested, showing that there was no difference in progression-free survival dependent on the sequence of the two drugs [17, 18]. A protocol of sequential alternating treatment regimen of tyrosine kinase inhibitor and chemotherapy was studied in non-small cell lung cancer [19]. Since there are no other treatment options than sorafenib and levatinib led to the hypothesis that alternating the use of sorafenib or lenvatinib may be a better and more effective way of treating refractory thyroid cancer than using one single agent alone.

In this study, we delineated the mechanism of drug resistance of cancer cells by inhibiting FGFR signaling and EMT in response to current treatments and discussed how these problems are being addressed.

## Methods

### Patients/tissue specimens

Fresh tumors were gained from patients with biochemical and histologically proven aggressive RAI-refractory papillary thyroid cancer who were treated at the Thyroid Cancer Center, Gangnam Severance Hospital, Yonsei University College of Medicine, Seoul, Korea. Further protocol and details are described in our previous articles [20, 21].

### Tumor cell isolation and primary culture

After resection, the tumors were transported to the laboratory. Normal tissue and fat were eliminated and rinsed with 1× Hank’s balanced salt solution (HBSS). Additional protocol and details are indicated in our previous article [21]. The research protocol was approved by the Institutional Review Board of the Thyroid Cancer Center, Gangnam Severance Hospital, Yonsei University College of Medicine (IRB Protocol: 3–2016-0076).

### Cell culture

The patient-derived aggressive papillary thyroid cancer (PTC) cell lines were grown in RPMI-1640 medium with 10~ 15% FBS. Authentication of the cell lines were carried out by with Cell ID system (Promega, Corporation, Madison, WI, USA) comparing their profiles with those published on the DMSZ database. Mycoplasma contamination was invariably checked with the Lookout Mycoplasma PCR Detection Kit (Sigma-Aldrich; MP0035).

### Cell viability assay

Cell proliferation was measured using the 3-(4, 5-dimethylthiazol-2-yl)-2, 5-diphenyl tetrazolium bromide (MTT) assay. Additional protocol is described in our previous article [20].

### SoLAT (Sorafenib Lenvatinib alternating treatment)

#### In vitro

At first, the combination treatment of the Sorafenib and Lenvatinib for 5 days, after Sorafenib and Lenvatinib alternating treatment for 5 days.

#### In vivo

At first, the combination treatment of the Sorafenib and Lenvatinib for 10 days, after Sorafenib and Lenvatinib alternating treatment for 10 days.

### Immunofluorescence analysis and confocal imaging

β-catenin expression was analyzed with immunofluorescent staining. Further protocol and data analysis details are described in our previous article [20].

### Immunoblot analysis

Antibodies against Ki-67 (Abcam), cyclin D1 (Santa Cruz Biotechnology, Dallas, TX, USA), CDK4 (Santa Cruz Biotechnology), p21 (Santa Cruz Biotechnology), p53 (Santa Cruz Biotechnology), p-ERK 1/2 (Santa Cruz Biotechnology), ERK 1/2 (Santa Cruz Biotechnology), Apaf-1 (Abcam), p-NFκB (Santa Cruz Biotechnology), Bcl-2 (Santa Cruz Biotechnology), caspase 3 (Santa Cruz Biotechnology), vimentin (Abcam), E-cadherin (Abcam), Snail (Abcam), Zeb1 (Abcam), and β-actin (Santa Cruz Biotechnology) overnight at 4 °C.

### Flow cytometry analysis of the cell cycle

Cells were treated with sorafenib and lenvatinib alone in an alternating regimen (SoLAT) in RPMI-1640 medium containing 10% FBS for 40 h, harvested by trypsinization, and fixed with 70% ethanol. Further protocol and data analysis details are described in our previous articles [20, 21].

### Tube formation assay

Human umbilical vein endothelial cells (HUVECs) (7 × 10^4^) were cultured with growth factor-reduced Matrigel (BD Biosciences, San Jose, CA, USA) in MV1 for 1 h for cell attachment, following which the endothelial growth basal medium-2 (EBM-2) was replaced with conditioned medium and cell culture was continued for 24 h. Tube length was quantified after 8 h by measuring the total cumulative tube length in 3 random microscopic fields with a computer-assisted microscope using Image J software. The original magnification used was × 100.

### Human thyroid cancer cell xenografts

The patient-derived aggressive PTC cells (3.5 × 10^6^ cells/mouse) were cultured in vitro and then injected subcutaneously into the upper left flank region of female BALB/c nude mice. After 11 days, tumor-bearing mice were grouped randomly (*n* = 10/group) and 10 mg/kg lenvatinib (administered p.o.) and 40 mg/kg sorafenib (administered p.o.), or lenvatinib or sorafenib alone (administered p.o.) were administered once every two days. Tumor size was measured on alternative days using calipers.. All experiments were approved by the Animal Experiment Committee of the Yonsei University.

### Immunohistochemistry

Primary Antibodies against p21 (Santa Cruz Biotechnology) and Bcl-2 (Abcam) were diluted with PBS at a ratio of 1:100 and incubated overnight at 4 °C. All tissue sections were counterstained with hematoxylin, dehydrated, and mounted.

### Statistical analysis

Statistical analyses were performed using GraphPad Prism software (GraphPad Software Inc., La Jolla, CA, USA). Immunohistochemistry results were subjected to ANOVA followed by a Bonferroni post hoc test. Values are expressed as the mean ± SD. *P* < 0.05 indicated statistical significance.

## Results

### Sorafenib and lenvatinib TKIs do not completely inhibit the proliferation of patient-derived PTC cells

**Fig. 1:**
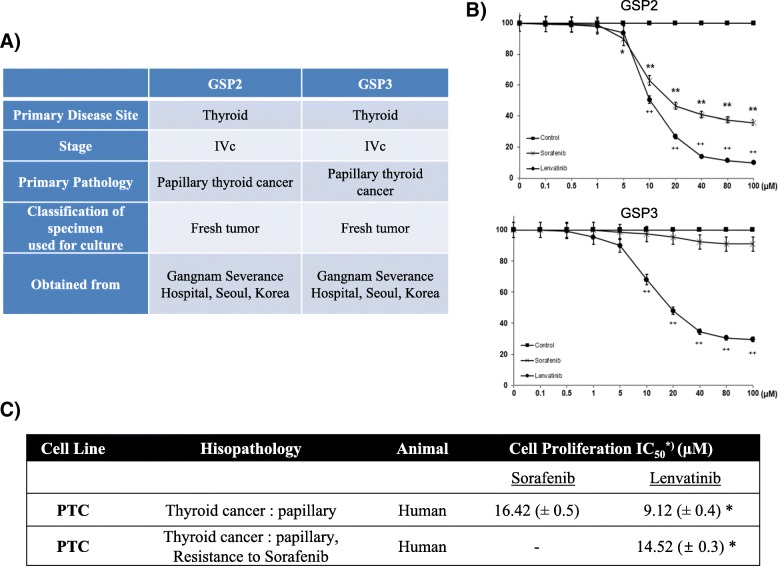
SoLAT (Sorafenib Lenvatinib Alternating Treatment) suppressed proliferation of GSP2 (patient-derived papillary thyroid carcinoma cells). **a** Information on the secondary established PTC cell line GSP2 and GSP3. **b** MTT assay for analysis of cell proliferation presence of sorafenib and lenvatinib. **c** Estimation of IC_50_ of the PTC cell line treated with sorafenib and lenvatinib

To investigate the anti-cancer effects of the TKIs sorafenib and lenvatinib on patient-derived PTC cells, we assayed GSP2 and GSP3 (Fig. 1a) cell proliferation in the presence of these compounds by the MTT assay (Fig. 1b). Concentration-dependent inhibition was not complete, although it was sufficient to determine the IC_50_ of sorafenib and lenvatinib in GSP2 and GSP3 cells (Fig. 1c).

### SoLAT is more effective than individual treatment in tumor suppression

**Fig. 2:**
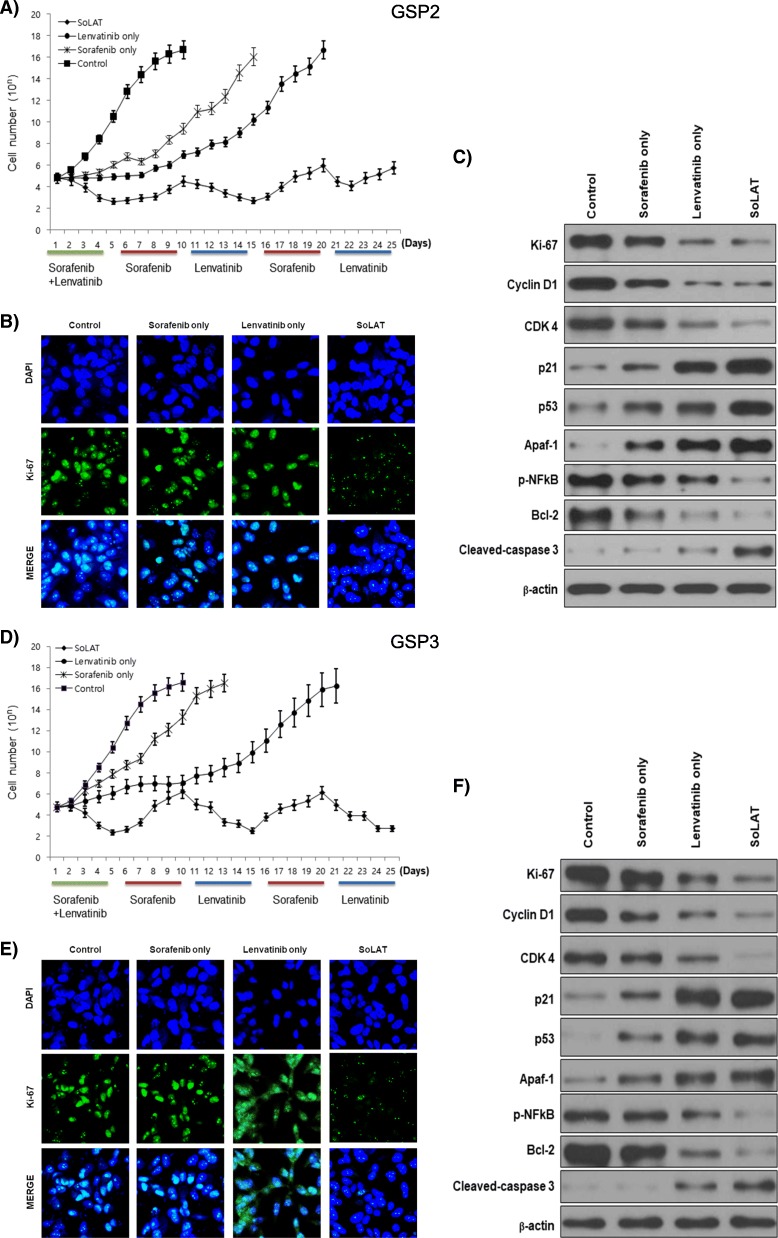
SoLAT was more efficiently induced cell cycle arrest and reduced anti-apoptotic factor than each treated groups on GSP2 and GSP3. **a** and **d** Anti-cancer activity of SoLAT on the advanced PTC cell line. Sorafenib Lenvatinib alternating treatment SoLAT was more efficient than the individual treatments. **b** and **e** Immunofluorescence staining for Ki-67. The SoLAT group suppressed Ki-67 expression. **c** and **f** Immunoblot analysis of cell-cycle arrest and apoptotic proteins showed marked increase in the levels of p21, p53, Apaf-1, and cleaved caspase 3, and decrease in the levels of Ki-67, cyclin D1, CDK 4, p-NFkB, and Bcl-2 in the SoLAT group than in the individual sorafenib or lenvatinib treatment groups

We investigated the anti-cancer activity of alternating treatment of sorafenib and lenvatinib (SoLAT) on advanced PTC. Individual treatments with sorafenib or lenvatinib did not significantly inhibit advanced PTC. SoLAT was more effective than the individual treatments (Fig. 2a and d). We performed immunofluorescence (Fig. 2b and e) and immunoblot analyses of cell cycle markers such as Ki-67 to confirm this observation (Fig. 2c and f). Sorafenib or lenvatinib treatment alone did not show any significant difference in Ki-67 levels compared to the control group. However, SoLAT suppressed Ki-67 expression (Fig. 2b and e). Evaluation of cell cycle-related protein levels by immunoblot analysis yielded similar results (Fig. 2c and f). A marked increase in the levels of p53, p21 (well-known inhibitors of the cell cycle), Apaf-1, and cleaved caspase 3 (pro-apoptotic factors) and decrease in the levels of cyclin D1, CDK 4, CDK 6 (positive regulators of the cell cycle), p-NFκB, and Bcl-2 (anti-apoptotic factors) were observed compared to sorafenib or lenvatinib treatment alone. These results conclusively show that cancer cell proliferation was inhibited by SoLAT.

### SoLAT is more efficient than the individual treatments in inducing cell cycle arrest, although it also increases drug resistance in advanced PTC

SoLAT increased the sub-G_0_/G_1_ population (*P* < 0.05) and induced cell death in advanced PTC, GSP2 and GSP3 (Table 1). Thus, the synergistic effect of sorafenib and lenvatinib potently induced the sub-G_0_/G_1_ population, leading to apoptosis, cell cycle arrest, and strong inhibition of advanced PTC viability. However, a small increase in the sub-G_0_/G_1_ population was observed after SoLAT, indicating that drug resistance was also induced by this treatment in advanced PTC.

**Table 1:** Cell cycle analysis: Alternating treatment with sorafenib and lenvatinib SoLAT showed significant increase in the sub-G_0_/G_1_ population and induction of cell death in advanced PTC (GSP2 and GSP3)

Status		Sub-G_0_G_1_	G_0_G_1_	S	G_2_/M
GSP2					
Control		1.4 ± 0.05	49.5 ± 0.05	27.2 ± 0.09	21.9 ± 0.05
Sorafenib only		4.7 ± 0.08	51.3 ± 0.07	25.4 ± 0.09	18.6 ± 0.05
Lenvatinib only		9.8 ± 0.04	49.6 ± 0.03	27.4 ± 0.12	13.2 ± 0.04
SoLAT	S + L	29.8 ± 0.09	46.7 ± 0.15	18.9 ± 0.05	4.6 ± 0.03
	Sorafenib	7.5 ± 0.02	49.5 ± 0.07	30.6 ± 0.01	12.4 ± 0.08
	Lenvatinib	21.9 ± 0.04	50.1 ± 0.12	22.3 ± 0.05	5.7 ± 0.02
	Sorafenib	1.7 ± 0.07	49.3 ± 0.04	28.5 ± 0.05	20.5 ± 0.09
	Lenvatinib	13.7 ± 0.15	48.3 ± 0.02	27.5 ± 0.01	10.5 ± 0.02
GSP3					
Control		1.1 ± 0.03	49.8 ± 0.08	27.5 ± 0.15	21.6 ± 0.12
Sorafenib only		2.4 ± 0.04	51.6 ± 0.06	26.4 ± 0.05	19.6 ± 0.01
Lenvatinib only		13.6 ± 0.04	53.2 ± 0.03	24.5 ± 0.07	8.7 ± 0.05
SoLAT	S + L	36.2 ± 0.15	45.6 ± 0.14	14.3 ± 0.19	3.9 ± 0.22
	Sorafenib	2.7 ± 0.15	51.2 ± 0.12	26.7 ± 0.12	19.4 ± 17
	Lenvatinib	32.4 ± 0.11	49.5 ± 0.21	13.5 ± 0.07	4.6 ± 0.15
	Sorafenib	2.4 ± 0.15	52.8 ± 0.25	27.6 ± 0.14	17.2 ± 0.13
	Lenvatinib	22.9 ± 0.24	55.1 ± 0.13	15.5 ± 0.19	6.5 ± 0.15

### SoLAT reduces EMT-mediated drug resistance in advanced PTC

**Fig. 3:**
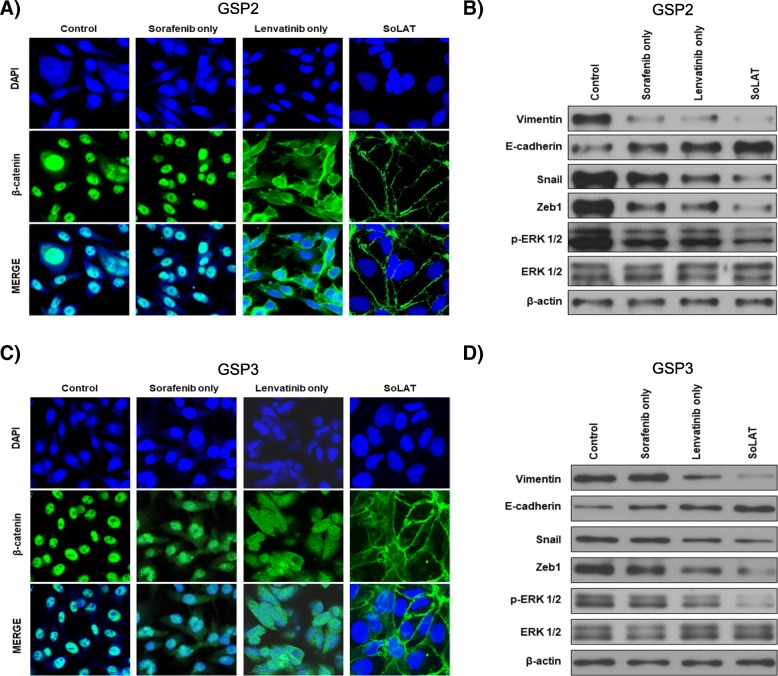
SoLAT prevents EMT through repression of β-catenin nuclear translocation in GSP2 and GSP3. **a** and **c** Immunofluorescence assay for nuclear translocation of β-catenin. Results confirmed that SoLAT inhibited nuclear translocation of β-catenin in the advanced PTC cells more potently than either agent alone. **b** and **d** Immunoblot analysis of EMT markers showed that most EMT markers such as vimentin, E-cadherin, Snail, and Zeb1 were inhibited by FGFR inhibition (p-ERK1/2) in the SoLAT group

Lenvatinib is well-known for reducing drug resistance-associated EMT by inhibiting FGFR. However, the patient-derived advanced PTC cells used in this study showed high levels of drug resistance. Consequently, no significant inhibition of drug resistance was achieved by individual sorafenib and lenvatinib treatments. However, SoLAT reduced the level of certain EMT markers. Immunofluorescence assay confirmed that SoLAT inhibited nuclear translocation of β-catenin in advanced PTC cells more potently than either agent alone (Fig. 3a and c). In addition, the levels of most EMT markers (vimentin, E-cadherin, Snail, and Zeb1) were reduced by FGFR inhibition (p-ERK 1/2) in the SoLAT group (Fig. 3b and d). This demonstrates that SoLAT effectively decreased EMT-mediated drug resistance via FGFR inhibition in advanced PTC.

### Angiogenesis of aggressive PTC is suppressed by SoLAT

**Fig. 4:**
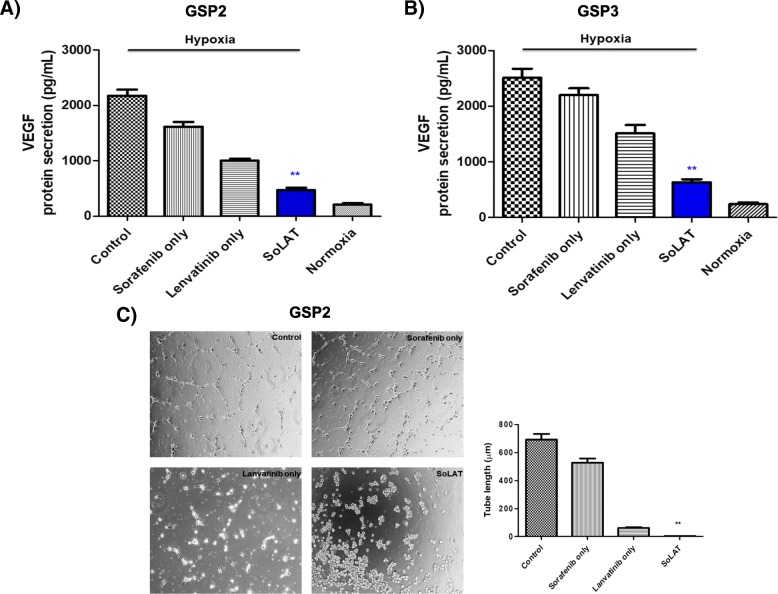
Secreted VEGFC expression and tube formation assay in conditioned media of indicated GSP2 and GSP3. **a** and **b** SoLAT suppressed VEGF secretion compared to that in the no treatment or single treatment group. **c** VEGF-induced tube formation assay of HUVECs showed that the tube length decreased in the SoLAT group

VEGF secretion activates angiogenesis, and SoLAT reduced angiogenic activity by suppressing VEGF secretion compared to the no treatment or individual treatment groups (Fig. 4a and b). Next, we used the VEGF-induced tube formation assay with HUVECs to analyze the effect of switching treatment on angiogenesis. HUVECs were cultured in the conditioned media of advanced PTC cells. After 16 h of culture, we evaluated the formation of a tubular structure with sorafenib or lenvatinib treatment alone and with SoLAT. The tube length in the advanced PTC-conditioned media alone was higher than that in the drug treatment groups (Fig. 4b). Quantitation of the tube length revealed that SoLAT in PTC-conditioned media considerably decreased the tube length compared to the no-treatment group and individual treatments (Fig. 4b). This demonstrated that secreted VEGF was inhibited by SoLAT.

### SoLAT induces tumor shrinkage in the xenograft model

**Fig. 5:**
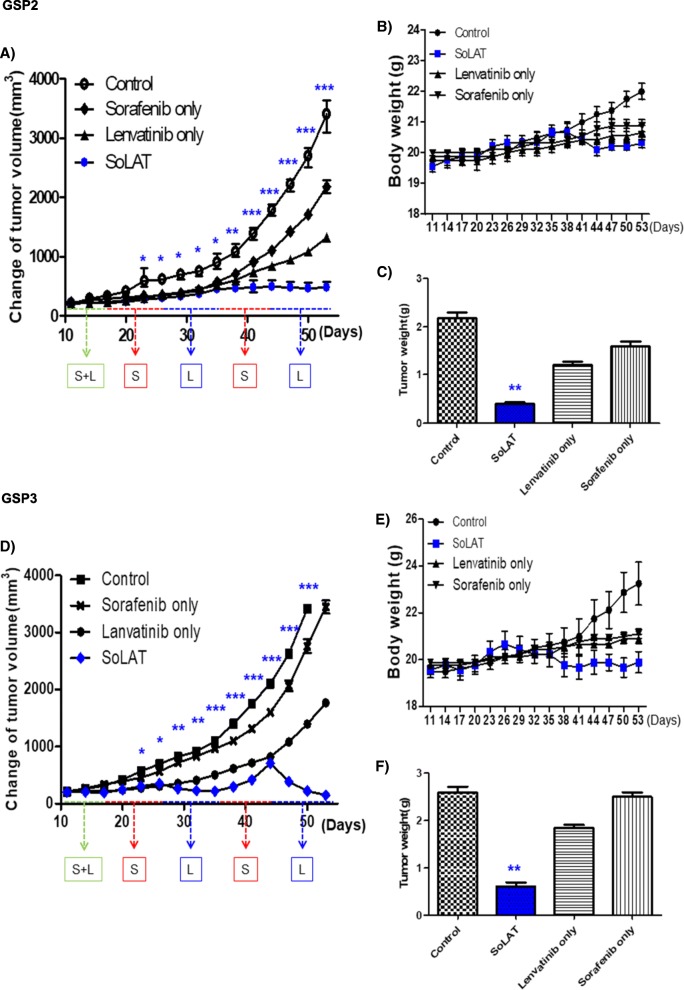
SoLAT was most efficiently induced tumor shrinkage in GSP2 and GSP3 xenografts model. **a** and **d** SoLAT suppressed tumor growth better than individual treatments with sorafenib or lenvatinib. **b** and **e** Sorafenib or lenvatinib treatment did not significantly affect the body weight of treated mice. No evidence of systemic toxicity or treatment-related death was observed in any group. **c** and **f** The SoLAT group showed significantly smaller tumor volumes compared to the individual treatment groups

**Fig. 6:**
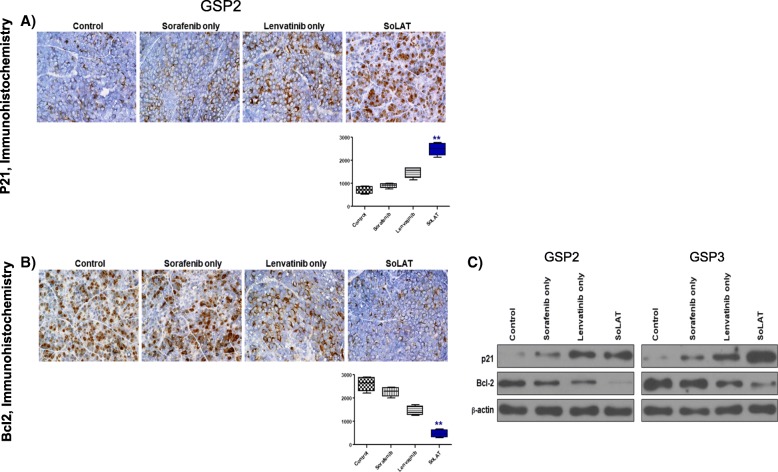
SoLAT was most efficiently induced cell cycle arrest and reduced anti-apoptotic factor on GSP2 and GSP3 xenografts model. **a** and **b** Immunohistochemistry showed that p21 levels were increased and whereas Bcl-2 levels were reduced by the alternating treatment with sorafenib and lenvatinib SoLAT. **c** Immunoblot analysis of cell-cycle arrest proteins showed marked increase in the levels of p21 and whereas anti apoptotic proteins were marked decrease in the levels of Bcl-2 by the SoLAT group than in the individual sorafenib or lenvatinib treatment groups on GSP2 and GSP3 xenografts model

Individual treatments with sorafenib or lenvatinib did not markedly suppress patient-derived aggressive PTC cell xenograft tumors; however, SoLAT significantly suppressed the proliferation of these tumors (Fig. 5a and d). No evidence of systemic toxicity or treatment-related death was observed in any group. Sorafenib or lenvatinib treatment did not significantly affect the body weight of mice (Fig. 5b and e). The SoLAT group showed significantly smaller tumor volumes than the individual sorafenib or lenvatinib treatment groups (Fig. 5c and f). Anti-apoptotic activity is a crucial factor for assessing the biological behavior of tumors. The levels of Bcl-2 and p21, which are common anti-apoptosis and cell cycle arrest markers, respectively, were determined by immunohistochemical and immunoblot analysis examination of patient-derived aggressive PTC cell xenograft tumors. SoLAT maximally decreased Bcl-2 and increased p21 levels (Fig. 6a, b and c). Thus, all of the results conclusively show that SoLAT exerts potent anti-cancer activity in the aggressive PTC cell xenograft model.

## Discussion

To our knowledge, this is the first study which showed that alternating treatment with the TKIs lenvatinib and sorafenib (SoLAT) was more effective than individual treatment with sorafenib or lenvatinib in inhibiting PTC tumor progression by inducing cell cycle arrest. There have been studies testing alternating chemoradiotherapy instead of concurrent chemoradiation for nasopharyngeal cancer and studies which showed alternating treatment for mantle cell lymphoma [22, 23]. Sequential use of two tyrosine kinase inhibitors was tested in metastatic renal cell carcinoma [17].

Studies using both in vitro cell culture and in vivo xenograft model provided evidence of tumor shrinkage in the alternative switching group. We suggest that these effects may be due to reduced EMT-mediated drug resistance in the aggressive PTC model.

EMT is induced in aggressive forms of thyroid cancer with elevated ZEB1 levels, which can promote drug resistance through EMT-dependent and EMT-independent mechanisms [24–26]. Studies have shown that downregulation of *ZEB1* could restore drug sensitivity [27, 28]. Sorafenib inhibits EMT in hepatocellular carcinoma, attenuates HGF secretion in polarized macrophages, decreases plasma HGF levels, and abolishes polarized macrophage-induced activation of the HGF receptor Met [29]. EMT reversal was shown to overcome drug resistance in lung adenocarcinoma [30].

The frequency and nature of adverse side effects differ between sorafenib and lenvatinib. Hand foot skin reaction is the most common side effect of sorafenib, whereas hypertension is the most common adverse effect of lenvatinib. Our results show that the IC_50_ is reduced when the drugs are used alternately compared to when used individually, which suggests that this can be an option for decreasing drug toxicity. In contrast to combination therapy, the drugs may be effective before being washed out of the body in the case of interval treatment; however, this requires further in vivo evidence. These findings may assist in developing a treatment protocol with reduced toxicity and enhanced drug efficacy.

TKIs are recommended for the treatment of RAI-refractory DTC patients with metastatic, rapidly progressive, symptomatic, and/or imminently threatening disease, which is not otherwise amenable to local control using alternative approaches. The benefits of systemic therapeutics have been demonstrated in the form of improved progression-free survival in three randomized, double-blinded, placebo-controlled clinical trials for vandetanib, sorafenib, and lenvatinib [6, 14, 15]. Sorafenib is known to inhibit RAF-1, a member of the RAF/MEK/ERK signaling pathway, and also BRAF, VEGFR-2, VEGFR-3, PDGFR-β, and c-KIT [31]. Lenvatinib has a potent inhibitory effect on VEGFR-2, VEGFR-3, PDGFRα/β, KIT, RET, and FGFR1–4. Lenvatinib differs from other drugs in its ability to inhibit FGFR1, providing efficacy in cases with VEGFR inhibitor resistance [16, 32, 33]. Despite favorable results in phase III trials and their status as the first line of treatment for RAI-refractory DTCs, both lenvatinib and sorafenib eventually elicit toxicity, and most patients discontinue them owing to unresponsiveness. A second-line kinase inhibitor therapy such as lenvatinib should be considered for patients with disease progression during initial kinase inhibitor therapy without prohibitive adverse effects [6].

Mechanisms for TKI resistance include receptor autophosphorylation, autophagy, involvement of hypoxia-inducing factor, epigenetic regulation, and EMT [34, 35]. Furthermore, several EMT-inducing cytokines such as TGF-β, FGF, HGF, insulin-like growth factor, and IL-6 may also be involved [30, 36]. We noted that SoLAT blocked constitutive ERK phosphorylation. The RAS/RAF/MEK/ERK signaling pathway is a major signaling pathway for EMT and metastasis, and inhibition of this pathway significantly reduces EMT [37]. Because we only analyzed ERK as a marker of this pathway in this study, other markers should be investigated in the future to confirm the FGFR inhibition-mediated EMT-reducing effects of lenvatinib.

It is necessary to investigate the mechanism underlying the success of the alternating treatment with lenvatinib and sorafenib compared to that of individual lenvatinib treatment in inhibiting the growth of aggressive PTC both in vitro and in vivo. Further studies are required to compare the efficiencies and toxicities of combination therapy and SoLAT. In addition, the effect of reusing one drug after development of resistance to both drugs (as an alternating therapy or combination therapy) has to be determined. Similarly, the effectiveness of reusing a therapeutic agent after development of resistance versus alternative interval treatment for prolonging disease-free survival has to be evaluated; in addition, the appropriate interval should be determined if interval treatment is found to be more efficient.

## Conclusions

The current study suggests that SoLAT was more effective than individual treatment with sorafenib or lenvatinib in inhibiting PTC progression by inducing cell cycle arrest and reducing EMT-mediated drug resistance.

## Abbreviations

EMT: epithelial mesenchymal transition
FGF: fibroblast growth factor
PTC: Papillary thyroid cancer, lenvatinib, sorafenib
TKI: tyrosine kinase inhibitor
